# Effect of Different Silane Coupling Agents on Properties of Waste Corrugated Paper Fiber/Polylactic Acid Composites

**DOI:** 10.3390/polym15173525

**Published:** 2023-08-24

**Authors:** Mannan Yang, Jian Su, Yamin Zheng, Changqing Fang, Wanqing Lei, Lu Li

**Affiliations:** 1 School of Mechanical and Precision Instrument Engineering, Xi’an University of Technology, Xi’an 710048, China; ymannan@163.com; 2 Faculty of Printing, Packaging Engineering and Digital Media Technology, Xi’an University of Technology, Xi’an 710054, China; sujian@xaut.edu.cn (J.S.); 18437959950@163.com (Y.Z.); lwq900529@163.com (W.L.); 3 Key Laboratory of Auxiliary Chemistry and Technology for Chemical Industry, Ministry of Education, Shaanxi University of Science and Technology, Xi’an 710021, China; lilu@sust.edu.cn; 4 Shaanxi Collaborative Innovation Center of Industrial Auxiliary Chemistry and Technology, Shaanxi University of Science and Technology, Xi’an 710021, China

**Keywords:** waste corrugated paper, polylactic acid, silane coupling agent, interface modifier

## Abstract

The surface of plant fibers was modified by silane coupling agents to prepare plant fiber/polylactic acid (PLA) composites, which can improve the dispersion, adhesion, and compatibility between the plant fibers and the PLA matrix. In this work, three silane coupling agents (KH550, KH560, and KH570) with different molecular structures were used to modify the surface of waste corrugated paper fibers (WFs), and dichloromethane was used as the solvent to prepare the WF/PLA composites. The effects of different silane coupling agents on the microstructure, mechanical properties, thermal decomposition, and crystallization properties of the composites were studied. The mechanical properties of the composites treated with 4 wt% KH560 were the best. Silane coupling agents can slightly improve the melting temperature of the composites, and WFs can promote the crystallization of PLA. The modification of WFs by silane coupling agents can increase the decomposition temperature of the WF/PLA composites. The content and type of silane coupling agent directly affected the mechanical properties of the WF/PLA composites. The interfacial compatibility between the WFs and PLA can be improved by using a silane coupling agent, which can further enhance the mechanical properties of WF/PLA composites. This provides a research basis for the further improvement of the performance of plant fiber/PLA composites.

## 1. Introduction

The shortage of global resources and the aggravation of environmental pollution make the balanced development between ecological protection and economic development very important. The development of green, economical, and recyclable new materials as well as their application have become the main research objectives of researchers. The conversion of renewable raw materials into biodegradable polymer materials to obtain environmentally friendly functional materials is a hot area of research in materials science [1,2,3]. Biodegradable polymer material can be decomposed into innocuous substances such as water and CO_2_ after a period of reaction under the action of microorganisms and catalytic enzymes. Polylactic acid (PLA), as a widely used and well-known biodegradable polymer material, has the advantages of good biocompatibility and biodegradability [4,5]. However, PLA has the disadvantage of a poor toughness, which limits the application of PLA in various fields [6,7]. Therefore, it is usually necessary to enhance PLA to improve its application performance. Many researchers have used chemical and physical modifications, including the incorporation of functional monomers with different molecular structures. Couture et al. [8] prepared two kinds of unidirectional flax composites based on PLA. The results showed that the tensile properties of flax/PLA and flax paper/PLA composites were similar to those of glass fiber fabrics impregnated with epoxy resin. Compared with unreinforced resin, the impact strength of flax paper/PLA composites was also higher. Faludi et al. [9] used wood fibers with a large aspect ratio as the raw materials to prepare PLA composites using an internal mixing method. It can be seen from the microscopic morphology of the cross section that when the fiber content reaches a certain level, the deformation and failure mechanism changes, which is due to the weak force between the fibers. The use of plant fibers to improve PLA can reduce its preparation cost and improve its strength and stiffness. Huda et al. [10] used kenaf fibers as a reinforcing agent and PLA as a matrix to prepare a laminated composite with good mechanical and thermo-mechanical properties. Natural-plant-fiber-reinforced composites have excellent mechanical properties and environmental protection characteristics.

The use of natural plant fibers and degradable materials to prepare composites can reduce the cost of composites and solve the problem of environmental pollution caused by non-degradable plastic waste [11]. There are still some problems, such as a poor high-temperature resistance and the limited toughening effect of natural plant fiber/PLA composites. It is still necessary to explore efficient methods to further improve the properties of plant fiber/PLA composites [12]. However, a large number of polar groups on the surface of the plant fibers makes it easy for them to agglomerate in the matrix, and they have a poor adhesion ability with PLA. Generally, it is necessary to improve the interfacial compatibility between natural plant fibers and PLA by surface modification to further improve the properties of plant fiber/PLA composites.

The common surface modification methods of natural plant fibers include physical modification (steam blasting pretreatment [13], heat treatment [14], or plasma treatment [15]) and chemical modification (alkali treatment [16], coupling agent treatment [17], graft copolymerization [18], acetylation treatment [19], etc.). Among these modification methods, coupling agent modification is an important chemical modification method. Coupling agents are divided into silanes, maleic anhydrides, isocyanates, and phthalates, and they have an important effect on improving the interfacial bonding strength of plant fibers and a PLA matrix. Avci et al. [20] investigated the influence of a coupling agent treatment on flax-fiber-reinforced PLA composites. The results showed that the coupling agent had a favorable effect on the thermal, thermo-mechanical, and mechanical properties of the composites. Treating plant fibers with a silane coupling agent does not reduce the mechanical properties of the fibers themselves, but improves the interfacial compatibility of the composites [21]. Mohd Ghazali et al. [22] found that the interfacial bonding of composites with fibers treated using silane and peroxide and composites coupled with MA-g-PLA noticeably improved, as supported by lower swelling indices and higher tensile strengths. Chen et al. [23] used four different silane coupling agents to improve the performance of wheat straw/polylactic acid composites. The results showed that the blending and tensile strengths of the composites were effectively enhanced, with the KH-570-modified composite exhibiting the best blending and tensile strengths. Wang et al. [24] used a silane coupling agent to modify the cellulose extracted from water bamboo byproducts to prepare polylactic PLA/cellulose antibacterial packaging material. The addition of a silane coupling agent improved the interfacial compatibility between the cellulose and the PLA. Nandi et al. [25] used two kinds of silane coupling agents with different mass fractions (0.5%, 1.0%, 2.5%, and 5.0%) to improve the reinforcement-matrix interfaces for developing nettle/PLA biocomposites with enhanced mechanical, thermo-mechanical, and biodegradation properties. The results showed that the interfacial strength of the biocomposite depends on the functional moiety and the level of concentration of the silane coupling agents. A silane coupling agent is called a molecular bridge, which connects hydrophilic and hydrophobic materials through hydrogen bonds or chemical bonds. The main mechanism is that the silane molecule contains bifunctional groups, some of which can react with the surface of the plant fiber to form chemical bonds, and the other part reacts with the polymer matrix to form chemical bonds, thus forming a bridge between the natural fiber and the polymer matrix. The main reaction process of silane coupling agent treatments of plant fibers includes hydrolysis, condensation, and the formation of hydrogen bonds and covalent bonds. Silane coupling agents are liquids and are easily mixed with different reinforcing fillers, which can be used to improve the compatibility of plant fibers and a polymer matrix [26,27].

Plant fibers such as ramie fibers, sisal fibers, bamboo fibers, hemp fibers, coconut shell fibers, straw fibers, kenaf fibers, and so on are the most widely used reinforcing fillers for preparing plant fiber/PLA composites. Many researchers have used plant fibers to reinforce bio-based composites, and they have studied the mechanical properties, thermal properties, interface properties, and degradation properties of the prepared composites [28,29,30,31,32,33]. The main raw materials of paper are plant fibers. The structure and properties of waste paper fibers are similar to those of natural plant fibers, and the extraction process is simpler. It is a promising way to use waste paper for the preparation of composite materials. Su et al. [34] found that waste corrugated paper fibers (WFs) can obviously improve the tensile strength, flexural strength, and deformation resistance of WF/PLA composites, and WFs can be uniformly dispersed in the PLA matrix by a solvent.

In recent years, with the rapid development of the ecommerce industry, the pollution problem of express corrugated paper packaging has become more and more serious. The question of how to control the environmental pollution caused by express packaging has become a research focus. The recycling of express packaging waste has become one of the important measures to solve the problem of waste packaging pollution [35,36,37]. Using WFs as the reinforcing filler of polymer matrix composites is beneficial for the protection of the ecological environment and the recycling of renewable resources.

In this work, three silane coupling agents (KH550, KH560, and KH570) with different molecular structures were used as interfacial modifiers to modify the surface of WFs, and dichloromethane was used as the solvent to prepare WF/PLA composites. The effects of different silane coupling agent additions on the microstructure, section structure, functional groups, mechanical properties, thermal decomposition process, and crystallization properties of the composites were studied.

## 2. Materials and Methods

### 2.1. Experimental Materials and Instruments

B-type corrugated boxes were collected from an express recycling point. PLA [34] (1.246 g/cm^3^, 100 mesh powder, melt flow index of 8 g/10 min (190 °C, 2.16 kg), Dongguan Yingsheng Plastic Chemical Co., Ltd., Dongguan, China), dichloromethane (CH_2_Cl_2_, analytical reagent, Nanjing Chemical Reagent Co., Ltd., Nanjing, China), anhydrous ethanol (C_2_H_6_O, analytical reagent, Shanghai Yinxiang Biotechnology Co., Ltd., Shanghai, China), deionized water (Xi’an Chemical Reagent Factory, Xi’an, China), KH550 (C_9_H_23_NO_3_Si, analytical reagent, Dongguan Dinghai Plastic Chemical Co., Ltd., Dongguan, China), KH560 (C_9_H_20_O_5_Si, analytical reagent, Dongguan Dinghai Plastic Chemical Co., Ltd., Dongguan, China), and KH570 (C_10_H_20_O_5_Si, analytical reagent, Dongguan Dinghai Plastic Chemical Co., Ltd., Dongguan, China) were obtained.

The experimental apparatuses and instruments used in this experiment are as follows: a Valley beater (IMT-VL01, Dongguan International Material Tester Co., Ltd., Dongguan, China), a constant-speed electric mixer (JJ-1B, Jintan Xicheng Xinrui Instrument Factory, Changzhou, China), an electric thermostatic water bath (DZKW-D-1, Shanghai Ke Heng Industrial Co., Ltd., Shanghai, China), an injection molding machine (TY-7003, Jiangsu Tian Yuan Test Instrument Co., Ltd., Yangzhou, China), a constant-temperature blast drying oven (101-4A8B, Beijing Kewei Yongxing Instrument Co., Ltd., Beijing, China), and an electronic balance (CPA225D, Sartorius AG, Gottingen, Germany).

### 2.2. Extraction of Waste Corrugated Paper Fibers

The B-type waste corrugated boxes were cut into cartons of 2 cm × 2 cm after removing the tape and non-adhesive labels. After soaking in deionized water for 24 h, the cartons were beaten using a Valley beater for 30 min, and then the pulp was filtered and dried at 100 °C. The WFs were obtained by grinding the above dried pulp into flocculence. The content of hemicellulose, cellulose, lignin, and other impurities in the WFs was measured using the Van Soest cellulose determination method [38], and the resulting data represent the averages of three tests. As shown in Table 1, the lignin content was the highest.

### 2.3. Modification of WFs

A total of 378 mL of C_2_H_6_O was added to 42 mL of deionized water, and a certain amount of a silane coupling agent, KH550, KH560, or KH570, was dropped into the above solution. The solution was stirred for 5 min, and then 50 g (25 wt%) of WFs was added into the solution. The solution was stirred manually for 10 min and then covered with plastic wrap and reacted for 3 h [39]. The modified waste corrugated paper fibers (MFs) were prepared by drying the obtained product at 80 °C and grinding it into flocs using a grinding mill. The silane coupling agent, KH550, KH560, or KH570, was added at 2 wt%, 4 wt%, 6 wt%, or 8 wt% (the weight fractions were relative to the WFs) to prepare the MFs.

### 2.4. The Preparation Process of MF/PLA Composites

The prescriptions of the prepared MF/PLA composites are shown in Table 2. Each sample is named MF/PX-Y, where X represents the name of the silane coupling agent (5 is KH550; 6 is KH560; 7 is KH570), P is PLA, and Y represents the percentage of the coupling agent relative to the mass of MFs.

**Figure 1:**
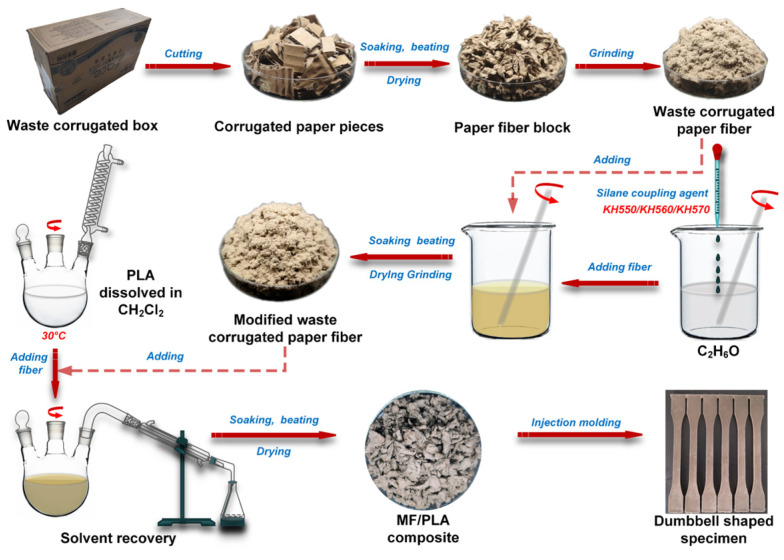
The preparation flowchart of MF/PLA composites.

PLA (150 g) was slowly added into 640 mL of the dichloromethane solvent, which was then heated in a water bath at 30 °C and stirred at 500 rpm for 4 h to completely dissolve the PLA. Then, WFs (or MFs) were added with a stirring speed of 800 rpm to disperse them completely. Finally, the obtained reaction solution was dried at 80 °C for 24 h, and the dichloromethane was collected using a condensation device. The obtained composites were completely dried and injected into dumbbell-shaped specimens, which were type A multipurpose specimens (ISO3167). The injection pressure was 57 MPa and the cooling time was 15 s. The mold was not heated and the temperatures of the injection molding machine were 140, 150, and 160 °C for the three heating zones. The process flow of the composites is shown in Figure 1.

### 2.5. Test and Characterization

The surface morphology of WFs and the tensile cross section of composites were characterized using a scanning electron microscope (SEM, SU-8010, Hitachi, Tokyo, Japan) with an accelerating voltage of 20 kV. The fiber diameter was measured using the Nano Measurer 1.2.5 software (Fudan University, Shanghai, China). The surface functional groups of WFs before and after modification were studied using a Fourier-transform infrared spectrometer (FT-IR, 8400S, Shimadzu, Kyoto, Japan) with a scanning range of 500–4000 cm^−1^ and a scan frequency of 40 times/s using KBr as the sample matrix. The tensile and bending strengths of the WF/PLA and MF/PLA composites were measured using a microcomputer-controlled universal testing machine (XWW-20A, Shanghai Jiezhun Instrument Equipment Co., Ltd., Shanghai, China) with a test speed of 5 mm/min, and the load was in the range of 0–10 kN. Each sample was measured 3 times, and the average value of the three was taken as the final test result according to the ISO 527-2 and ISO 178 [39] international standards. The thermal properties of the WF/PLA and MF/PLA composites were studied using a differential scanning calorimeter (DSC200F3, NETSCH Group, Selb, Germany) in a N_2_ atmosphere with a gas flow of 50 mL/min. The temperature was first raised from 20 °C to 150 °C at a heating rate of 10 °C/min and held for 2 min at 150 °C, and was then cooled to 20 °C at a heating rate of 10 °C/min to eliminate the thermal history. Finally, the temperature was heated to 220 °C with a heating rate of 10 °C/min. The DSC crystallinity was calculated using the NETZSCH Proteus thermal analysis software, version 6.1.0 (NETZSCH-Geraetebau GmbH, Selb/Bayern, Germany). An X-ray diffractometer (XRD-7000, Shimadzu, Kyoto, Japan) was used to test the crystallization of the samples. The scanning range was 5–60° and the scanning speed was 10 °/min. The thermal stability of the WF/PLA and MF/PLA composites was analyzed using a thermogravimetric analyzer (TG209F3, NETSCH Group, Selb, Germany) with a heating rate of 10 °C/min from 30 °C to 600 °C under a N_2_ atmosphere with a gas flow of 50 mL/min.

## 3. Results and Discussion

### 3.1. Micromorphology of Modified Waste Corrugated Paper Fibers

**Figure 2:**
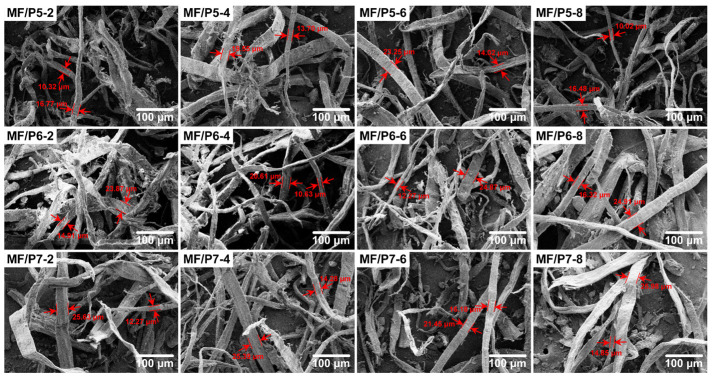
The morphology of modified waste corrugated paper fibers.

The plant fibers from corrugated paper contain cellulose, hemicellulose, lignin, and a small amount of silicate, and they have a rough surface. The surface roughness of the fibers is conducive to the formation of friction between the WFs and the PLA matrix, which can improve the mechanical properties of the composite [40]. The micromorphology of MFs is shown in Figure 2. It can be seen from the mark of the SEM images that the WFs modified by a silane coupling agent are mostly slender fibers with a width of 10–20 μm. The MFs are uniformly dispersed without an obvious agglomeration phenomenon, because they are coated with a layer of non-polar chain segments after the modification, which can weaken the hydrogen bonds between the WFs. There are no evident differences in the microstructures of WFs treated with the three coupling agents.

### 3.2. Tensile Section Morphology of MF/PLA Composite

**Figure 3:**
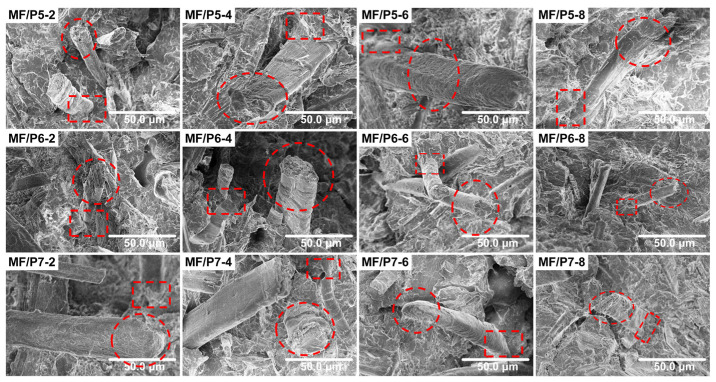
The tensile fracture surface morphology of MF/PLA composites.

The tensile fracture surface morphology of the MF/PLA composites is shown in Figure 3. It can be seen that the MFs are not entangled or agglomerated with each other, but uniformly dispersed in the PLA matrix. As shown by the elliptical frames in the figure, the fractured MF cross sections are accompanied by some debonding fibers, which were pulled out from the PLA matrix. Some of the PLA matrix is attached to the surface of the pulled MFs, indicating that the interface compatibility between the MFs and the matrix was good. The rectangular frames in the figure show that the interstices between the MFs and PLA matrix are small, and the boundary between them is blurred, indicating that, after the surface treatment, the compatibility between the WFs and the PLA matrix is good, and the binding force between the WFs and the PLA is enhanced [41]. The results indicate that the compatibility between the PLA and the waste paper fibers treated with a silane coupling agent is high, and the repulsive force between the molecular chains is also reduced [42].

### 3.3. FT-IR Analysis of Modified Waste Corrugated Paper Fibers

**Figure 4:**
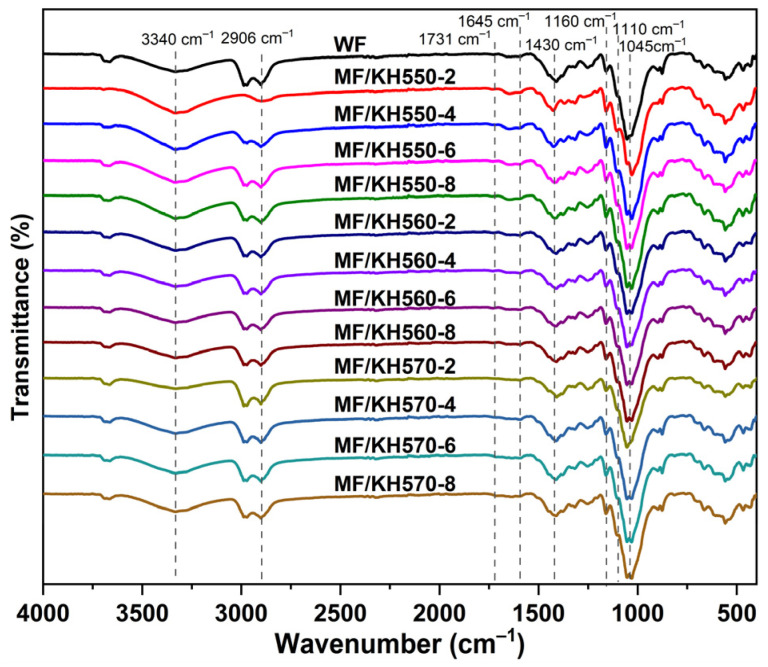
FT-IR spectra of MF/PLA composites.

The infrared spectrum of the MFs is shown in Figure 4. The sample was named MF/KHX-Y, where KHX represents the name of the silane coupling agent (X = 550, 660, or 770) and Y represents the percentage of the coupling agent relative to the mass of the MFs. The intensity of the characteristic MF peak at 1045 cm^−1^ is relatively strong, which is caused by the C=O stretching vibration of cellulose and hemicellulose. The characteristic peak at 1160 cm^−1^ was attributed to the stretching vibration of C-O-C in cellulose and hemicellulose. The characteristic peak at 1430 cm^−1^ was ascribed to the methylene (CH_2_) bending vibration of cellulose. The characteristic peak of cellulose at 1645 cm^−1^ is caused by the -OH stretching vibration of cellulose. The characteristic peak at 2906 cm^−1^ corresponds to the stretching vibration of methyl -C-H and -OH between the molecules in the WFs. The characteristic peak at 3340 cm^−1^ is caused by the stretching vibration peak of -OH.

The characteristic asymmetric stretching vibration peak of Si-O-C is at 1110 cm^−1^, which may be caused by the self-condensation between the silanol groups during the modification process. The stretching vibration peak of C=O is at 1731 cm^−1^, and the peak of the MFs is stronger than that of the WFs, which is due to the reaction between the coupling agent and the WFs [43]. The characteristic peak at 2906 cm^−1^ is obviously enhanced with an increase in the coupling agent content, indicating that the C-H structure in the MFs was increased. There is a stretching vibration peak from -OH in the WFs at 3340 cm^−1^ [44].

**Figure 5:**
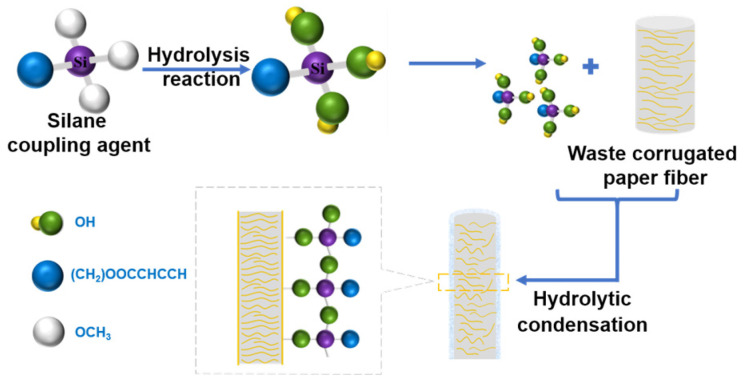
Reaction principle of WFs and silane coupling agent.

The reaction principle of the silane coupling agent and the WFs is shown in Figure 5. The methoxy group in the silane coupling agent undergoes a hydrolysis reaction in the presence of water to form a stable Si-O-C structure. Because silanol is extremely unstable, it is easy to react it with -OH on the surface of cellulose to shrink and act on the surface of the WFs. After the treatment with the silane coupling agent, the vibration peak is obviously strengthened with an increase in the amount of the coupling agent, which is caused by the hydrolysis reaction of the silane coupling agent. The silane coupling agent is hydrolyzed to produce -Si-OH, which then reacts with the -OH on the surface of the fibers through hydrogen bonding, dehydrates with the -OH to form an ether bond under heating conditions, and finally connects the other end of the coupling agent to the fiber to react with the -OH or -COOH of PLA. The changes in these characteristic peaks indicate that the coupling agent successfully grafted onto the WFs. The position and strength of the characteristic peaks of the WFs treated with the three silane coupling agents are similar, which is due to the three silane coupling agents having similar molecular structures and reaction mechanisms.

### 3.4. Mechanical Properties of MF/PLA Composites

**Figure 6:**
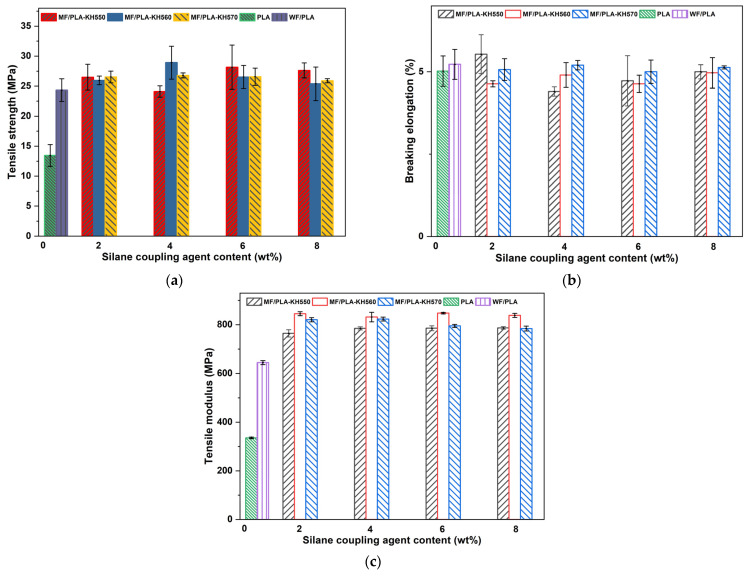
The mechanical properties of PLA and composites: (**a**) tensile strength, (**b**) breaking elongation rate, and (**c**) tensile modulus.

The tensile strength of the MF/PLA composites is shown in Figure 6a. It can be seen that the effects of the three silane coupling agents at different weight fractions on the tensile strength of the composites are obvious. After adding one of the three different coupling agents, the tensile properties of the composites obviously improved. With an increase in the amount of the coupling agent from 2 wt% to 8 wt%, the tensile strength of the composites increased first and then decreased. The silane coupling agent in a low amount could not completely modify the surface of the plant fibers, so the mechanical strength of the composites could not be effectively enhanced. When the amount of the coupling agent added was too large, due to the excessive amount of free coupling agent small molecules, the interfacial bonding force of the composites was affected, resulting in a decrease in the mechanical properties. It is very important to select the appropriate amount of the coupling agent.

When KH550 is used as the modifier, the tensile strength of the composite is the highest when the addition amount is 6 wt%, and is 109.0% higher than that of the pure PLA matrix. This is because the Si-OH in KH550 can form hydrogen bonds with the hydroxyl groups on the surface of the WFs, which can make the WFs and PLA combine tightly, thus improving the interfacial compatibility of the WFs and PLA and improving the mechanical properties of the WF/PLA composites. When KH560 is used as the modifier, the tensile strength of the composites is the best when the addition amount is 4 wt%, and is 115.0% higher than that of the pure PLA matrix. This is due to KH560 containing three methoxy groups, and the epoxy group in KH560 can play a good compatibility role with the PLA matrix. The Si-O-C generated after the hydrolysis of the methoxy group can react with the -OH on the surface of the WFs. When KH570 is used as the modifier, the tensile strength of the composite is the highest when the addition amount is 4 wt%, and is 99.2% higher than that of the pure PLA. The results are roughly consistent with the results of the elongation at break, which is shown in Figure 6b. The addition of a silane coupling agent increases the elongation at break of the composite material. This is because the addition of a silane coupling agent increases the intermolecular interaction force. Under the same conditions, the ability to withstand external forces during the stretching process increases, and the elongation at break of the composite material increases. It can also be seen that the elongation at break of the modified composite material is the largest when the addition amount of KH-550 is 2%. The interfacial bonding force of the composite material modified by the silane coupling agent increases. When it is stretched by an external force, a large deformation occurs before it can be broken, resulting in a large elongation at break. The addition of a silane coupling agent improves the flexural modulus of the polymer. As shown in Figure 6c, the tensile modulus of the composites is higher than that of PLA. The addition of a coupling agent increases the tensile modulus of the composites. Different weight fractions of the same coupling agent make little difference to the increase in the tensile modulus, and the tensile modulus of the KH560-modified composites increased the most. Therefore, the coupling agent can act on the WFs to enhance the binding force between the two and the interface bonding force, thereby improving the mechanical properties of the WF/PLA composite.

**Figure 7:**
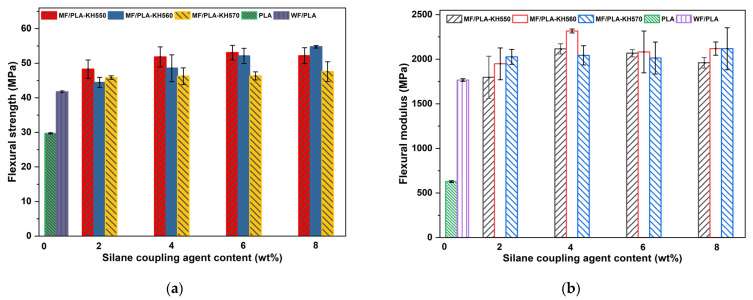
The mechanical properties of PLA and composites: (**a**) flexural strength and (**b**) flexural modulus.

The flexural strength of the MF/PLA composites is shown in Figure 7a. The surface treatment of the WFs by the coupling agent can obviously improve the flexural strength of the MF/PLA composites. With an increase in the amount of the coupling agent from 2 wt% to 8 wt%, the flexural strength of the composites shows an increasing trend.

When KH550 is used as the modifier, the flexural strength of the composite with 6 wt% KH550 is the highest, and is 78.4% higher than that of the pure PLA matrix. When KH560 is used as the modifier, the flexural strength of the composite with 8 wt% KH560 is the highest, and is 84.2% higher than that of pure PLA. When KH570 is used as the modifier, the flexural strength of the composite with 8 wt% KH570 is the best, and is 60.0% higher than that of the pure PLA. The flexural modulus is shown in Figure 7b; the addition of a silane coupling agent improves the flexural modulus of the polymer. When the addition amount of KH560 is 4 wt%, the flexural modulus is the largest. After the WFs are modified by a coupling agent, the interaction force between the non-polar chain segments on the WF surface and the PLA chains is enhanced, which improves the binding force between the WFs and the PLA matrix and is beneficial for improving the bending strength of the MF/PLA composites.

When the addition content of the silane coupling agent is 4 wt%, KH550 and KH570 can easily achieve self-polymerization, and the mutual condensation of silanol reduces the coupling activity of the silane interface obviously, which affects the effective bonding of the WFs and the PLA matrix interface. KH560 is less prone to self-polymerization and reducing the activity of siloxy, so the combination of siloxy and WFs is more effective than that of KH550 and KH570.

### 3.5. DSC Analysis of MF/PLA Composites

**Figure 8:**
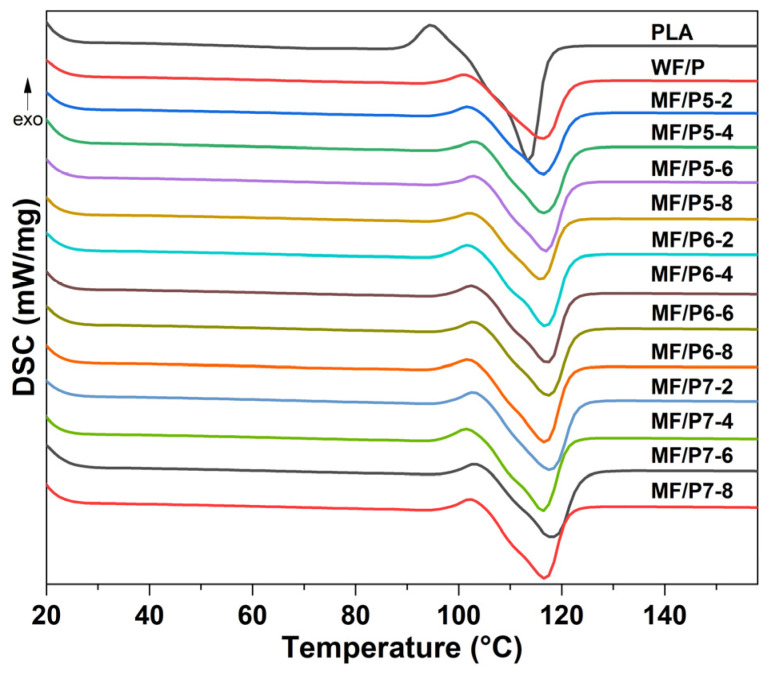
DSC curves of MF/PLA composites.

The DSC curves of the MF/PLA composites are shown in Figure 8. After the WFs are modified, the crystallization and melting temperatures of the MF/PLA composites show a relatively stable state, and the overall change is not obvious. The highest melting temperatures of the composites with the addition of MFs and treated with KH550, KH560, and KH570 are 116.77 °C, 117.29 °C, and 118.13 °C, respectively. Compared with the PLA matrix (113.76 °C), the melting temperature of the MF/PLA composites was slightly increased, but the overall change was not obvious, which is because the coupling agent makes the interface bonding tighter and hinders the movement of the PLA molecular chains. The addition of WFs treated with a silane coupling agent had no significant effect on the thermal properties of the composites. This is because the use of a silane coupling agent does not produce new substances in the composites. The crystallinity of PLA and the composites is shown in Table 3. The results show that the addition of WFs reduced the crystallinity of PLA, which is because the poor compatibility between WFs and PLA makes it difficult for PLA to nucleate, resulting in a lower crystallization rate and a lower crystallinity. Compared with an unmodified WF/PLA composite, the addition of an appropriate amount of a silane coupling agent increased the crystallization rate and improved the crystallinity of the composites. However, when the addition of the silane coupling agent increased, the crystallinity was smaller. The addition of a silane coupling agent promoted the formation of the nucleation center of PLA in the composites, promoted the development of single-oriented crystals, and improved the mechanical strength of the composites.

### 3.6. TG Analysis of MF/PLA Composites

**Figure 9:**
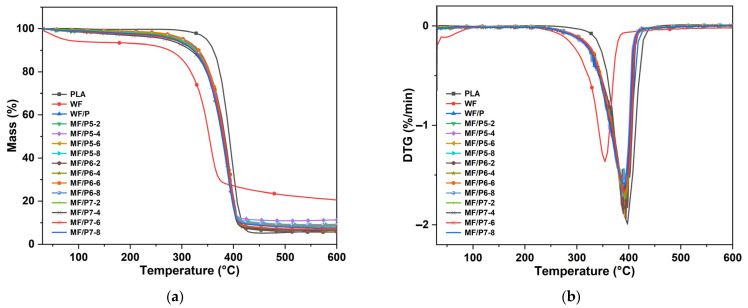
TG (**a**) and DTG (**b**) curves of MF/PLA composites.

The TG and DTG curves of the MF/PLA composites, WFs, and PLA are shown in Figure 9. Different types and weight fractions of the silane coupling agents used to modify the WFs had a slight effect on the thermal decomposition temperature of the composites. The main ingredients in WFs, cellulose, hemicellulose, and lignin, decompose between 285 and 378 °C. The thermal decomposition temperature of pure PLA is about 348 °C (5 wt% weight loss), and the temperature for the maximum decomposition rate is about 398 °C. In the thermal decomposition process of PLA, the macromolecular skeleton is broken, and the intramolecular transesterification produces oligomers and lactic acid molecules. The elimination reaction and free radical reaction produce small molecules such as carbon dioxide, ethanol, and mixed organic matter, which are carried out with the carrier gas flow [45]. When the addition amount of the silane coupling agent KH550 is 8 wt%, the thermal decomposition temperature of the MF/PLA composite is the lowest and is 8 °C lower than that of pure PLA, and the temperature corresponding to the maximum decomposition rate is 1 °C lower than that of pure PLA. When the addition amount of KH560 is 2 wt%, the thermal decomposition temperature of the composite is the lowest and is 8 °C lower than that of pure PLA, and the temperature corresponding to the maximum decomposition rate is 6 °C higher than that of pure PLA. When the addition amount of KH570 is 4 wt%, the thermal decomposition temperature of the composite is the lowest and is 22 °C lower than that of pure PLA, and the temperature corresponding to the maximum decomposition rate is 2 °C higher than that of pure PLA.

The addition of a coupling agent makes the temperature for the initial decomposition of the composite material increase and the temperature for the maximum decomposition rate increase slightly. This indicates that the addition of a coupling agent improves the interfacial compatibility of the MF/PLA, so the thermal stability shows a slight rising trend. The addition of a coupling agent improves the properties of the composites, and the slight increase in the thermal stability has little effect on the performance of the composites. Therefore, modifying the composites by adding a coupling agent is an effective method for improving the comprehensive properties of the composites.

### 3.7. XRD Analysis of MF/PLA Composites

**Figure 10:**
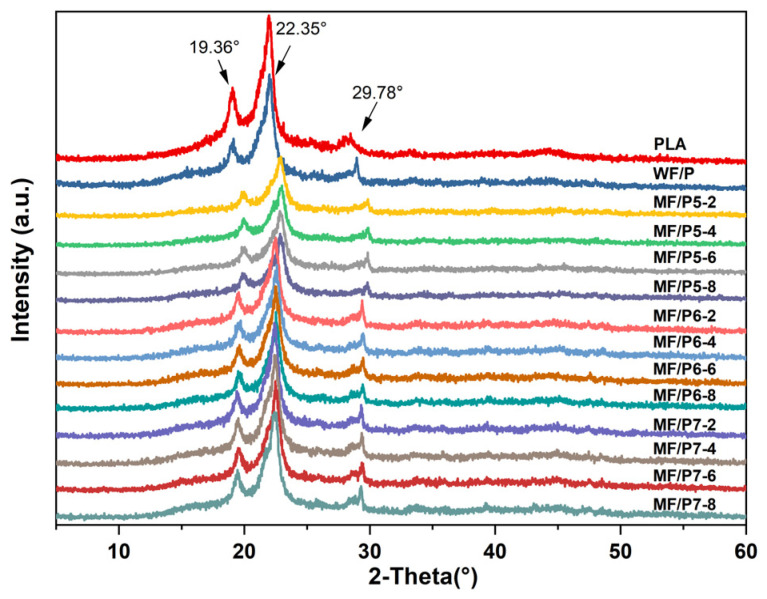
XRD pattern of WF/PLA and MF/PLA composites.

The XRD patterns of PLA and the composites are shown in Figure 10. The diffraction peaks at 19.36°, 22.35°, and 29.78° are consistent with the structure of PLA’s crystal diffraction peak [23]. The addition of a silane coupling agent did not change the original crystal form of PLA, but reduced the diffraction peak intensity compared with PLA. This is because the addition of a silane coupling agent enhanced the interaction between PLA and the WFs, increased the molecular chain entanglement density, hindered the transformation of molecular chains from disorder to order, and reduced the crystallinity of PLA. The diffraction peak intensity of the WF/PLA composites with the addition of MFs treated by KH560 or KH570 was not significantly weakened compared with the diffraction peak intensity of composites without coupling agent modification, which indicates that KH560 and KH570 have little effect on the crystallization properties of composites. However, the diffraction peak intensity of the composites with the addition of MFs treated by KH550 was significantly weakened, indicating that the addition of KH550 destroyed the crystal structure inside the composites.

## 4. Conclusions

WF/PLA and MF/PLA composites were prepared using the solvent method. The results show that the WFs and MFs are uniformly dispersed in the PLA matrix without agglomeration. The phase interface between the modified WFs and PLA becomes blurred, and the interfacial bonding force and dispersion state are improved. The enhancement effect of the MFs on the mechanical properties of the composites is far better than that of the WFs. The content and type of silane coupling agent have a great effect on the mechanical properties of the composites. Among them, the surface treatment effect of KH560 is better than that of KH550 or KH570. The mechanical properties of the composites treated with 4 wt% KH560 (MF/P6-4) are the best, and the tensile and flexural moduli are enhanced. The silane coupling agent is grafted to the WF surface by a condensation reaction with the hydroxyl group on the surface of the WFs. The addition of WFs and MFs decreases the crystallinity of PLA, but does not change the crystal form of PLA. Compared with the unmodified composite, fibers modified by a silane coupling agent improve the crystallinity of the composite, which can prove an improvement in the interfacial adhesion between the fibers and the PLA matrix, and the mechanical properties of the composites are further enhanced. In addition, different types and weight fractions of silane coupling agents have little effect on the thermal decomposition temperature of the composites.

## Figures and Tables

**Table 1:** Components of waste corrugated paper fibers (wt%).

Components	Lignin	Cellulose	Hemicellulose	Other
Content	42.02 ± 0.09	13.23 ± 0.08	20.18 ± 0.02	25.06 ± 0.15

**Table 2:** Prescriptions of the MF/PLA composites.

Silane Coupling Agent	Sample	Coupling Agent (wt%)	WF (wt%)	PLA (wt%)
KH550	MF/P5-2	2	25	75
	MF/P5-4	4	25	75
	MF/P5-6	6	25	75
	MF/P5-8	8	25	75
KH560	MF/P6-2	2	25	75
	MF/P6-4	4	25	75
	MF/P6-6	6	25	75
	MF/P6-8	8	25	75
KH570	MF/P7-2	2	25	75
	MF/P7-4	4	25	75
	MF/P7-6	6	25	75
	MF/P7-8	8	25	75
/	WF/P	/	25	75

**Table 3:** The crystallinity of PLA and the composites.

Sample	∆H_f_/(J/g)	X_c_/%
MF/P5-2	80.22	56.5
MF/P5-4	88.33	62.21
MF/P5-6	81.11	57.12
MF/P5-8	72.56	51.1
MF/P6-2	85.86	60.46
MF/P6-4	93.12	65.58
MF/P6-6	86.01	60.57
MF/P6-8	86.06	60.61
MF/P7-2	94.17	66.32
MF/P7-4	87.14	61.36
MF/P7-6	90.59	63.8
MF/P7-8	84.11	59.23
WF/P	75.7	53.31
PLA	127.6	89.84

## Data Availability

The data are contained within the article. The data presented in this study on the synthesis and properties of a carbon microsphere from waste office paper are available.
